# Melatonin protects against ischemic stroke by modulating microglia/macrophage polarization toward anti‐inflammatory phenotype through STAT3 pathway

**DOI:** 10.1111/cns.13261

**Published:** 2019-12-02

**Authors:** Zong‐Jian Liu, Yuan‐Yuan Ran, Shu‐Yan Qie, Wei‐Jun Gong, Fu‐Hai Gao, Zi‐Tong Ding, Jia‐Ning Xi

**Affiliations:** ^1^ Department of Rehabilitation Beijing Rehabilitation Hospital Capital Medical University Beijing China

**Keywords:** inflammation, ischemic stroke, melatonin, microglia/macrophage polarization

## Abstract

**Aims:**

Microglia and infiltrated macrophages play important roles in inflammatory processes after ischemic stroke. Modulating microglia/macrophage polarization from pro‐inflammatory phenotype to anti‐inflammatory state has been suggested as a potential therapeutic approach in the treatment of ischemic stroke. Melatonin has been shown to be neuroprotective in experimental stroke models. However, the effect of melatonin on microglia polarization after stroke and underlying mechanisms remain unknown.

**Methods:**

In vivo, cerebral ischemia was induced by distal middle cerebral artery occlusion (dMCAO) in C57BL/6J mice. Melatonin was injected intraperitoneally (20 mg/kg) at 0 and 24 hours after ischemia. In vitro, the microglial cell line BV2 was stimulated to the pro‐inflammatory state with conditioned media (CM) collected from oxygen‐glucose deprivation (OGD) challenged neuronal cell line Neuro‐2a (N2a). Real‐time PCR was utilized to detect the mRNA expression of microglia phenotype markers. Activation of signal transducer and activator of transcription 3 (STAT3) pathway was determined by Western blot of phosphorylated STAT3 (pSTAT3). A neuron‐microglia co‐culture system was used to determine whether melatonin can inhibit the neurotoxic effect of pro‐inflammatory microglia to post‐OGD neurons.

**Results:**

Melatonin treatment reduced brain infarct and improved neurological functions 3 days after dMCAO, which was accompanied by decreased expression of pro‐inflammatory markers and increased expression of anti‐inflammatory markers in the ischemic brain. In vitro studies confirmed that melatonin directly inhibited the pro‐inflammatory responses in BV2 cells upon exposure to OGD neuron CM. The microglia possessing pro‐inflammatory phenotype exacerbated post‐OGD N2a cells death, whereas melatonin reduced such neurotoxic effect. Further, melatonin enhanced the otherwise inhibited pSTAT3 expression in BV2 cells treated with OGD neuron CM. STAT3 blockade significantly reduced the effect of melatonin on microglial phenotype shift. Conclusion: Melatonin treatment ameliorates brain damage at least partially through shifting microglia phenotype from pro‐inflammatory to anti‐inflammatory polarity in a STAT3‐dependent manner.

## 1 INTRODUCTION

Stroke is one of the primary causes of death and disability worldwide. Ischemic stroke accounts for approximately 85% of total stroke. A large number of reports in the past few decades have shown that neuroprotective strategies target to only neurons fails to produce a better outcome after stroke.^1^, ^2^ Accumulating research demonstrates that cytokine‐dependent microenvironments play crucial roles in the progress of stroke.^3^ New therapeutic strategies toward the regulations of microenvironment to promote functional outcomes and tissue repair after stoke have recently attracted tremendous attention.

Inflammatory responses are one of the main pathophysiological processes involved in secondary injury after stroke.^4^ Microglia are resident immune cells in the central nervous system (CNS), contributing to the maintenance of CNS homeostasis in the normal condition. Microglia can drastically alter their phenotypes and functions (pro‐inflammation, anti‐inflammation and et al.) in response to microenvironmental changes.^5^, ^6^ The pro‐inflammatory phenotype expresses signature markers including CD86 and CD68 and tends to release destructive mediators such as tumor necrosis factor (TNF‐α), inducible nitric oxide synthase (iNOS), interleukin‐1β (IL‐1β), and nitric oxide (NO).^7^ In contrast, the anti‐inflammatory phenotype characterized by expression of molecules such as arignase1, Ym1/2, CD206, and produces beneficial mediators, including interleukin‐4 (IL‐4), interleukin‐10 (IL‐10), and transforming growth factor‐β (TGF‐β). Dual roles of microglia are observed at different stages after stroke.^7^, ^8^, ^9^ Microglia and infiltrated macrophage initially polarize toward a neuroprotective anti‐inflammatory phenotype after stroke, but gradually transform into a detrimental pro‐inflammatory phenotype. In vivo and vitro studies revealed that inhibiting pro‐inflammatory microglia phenotype and/or stimulating anti‐inflammatory microglia state were neuroprotective.^7^, ^10^, ^11^ For example, some drugs, including ginkgolide B,^10^ malibatol A,^11^ thiamet G,^12^ baicalein,^13^ isosteviol sodium,^14^ curcumin,^15^ oxytocin,^16^ and dimethyl fumarate^17^ have been reported to protect against brain injury through regulating microglia/macrophage polarization and inflammatory responses in experimental models of brain ischemia or hypoperfusion. Some physiological factors, such as hypothermia,^18^, ^19^, ^20^ also altered microglia/macrophage phenotype. Exploring novel approaches that shift microglia/macrophage phenotype might provide more therapeutic strategy for ischemic stroke.

Melatonin (*N*‐acetyl‐5‐methoxy‐tryptamine) is a hormone secreted by the pineal gland, and its principal function is to regulate the circadian rhythm in mammals. Melatonin has been implicated in diverse physiological processes including mood behavior, blood pressure regulation, ovarian physiology, and osteoblast differentiation.^21^ Its ability to readily cross the blood‐brain barrier (BBB) makes melatonin an ideal neuroprotective agent. A large number of studies have demonstrated the involvement of melatonin in a variety of disease models, including stroke, traumatic brain injury, Alzheimer's disease, and Parkinson's disease.^22^, ^23^, ^24^, ^25^ Administration of melatonin before or after cerebral ischemia has been shown to reduce infarct volume, enhance BBB integrity, and improve behavioral outcomes in animal models of stroke.^26^, ^27^ Over the past decades, melatonin has emerged as a very potent free radical scavenger and antioxidant. It was able to directly protect against stroke‐induced neuron cells death and ameliorate behavioral deficits by inhibition of oxyradicals and peroxynitrite production. Recently, an increasing number of studies have successively demonstrated that melatonin's neuroprotection against ischemic stroke derived from the inhibition of mitochondrial cytochrome C release [20] and the decrease of inflammatory responses.^28^ However, the effect of melatonin on microglia/macrophage responses after stroke remains to be elucidated.

In the present study, we used a mouse model of dMCAO to analyze the effect of melatonin on microglia/macrophage polarization and inflammatory response after stroke. In vitro studies revealed the cellular mechanism of melatonin in regulating microglia/macrophage polarization. A microglia‐neuron co‐culture system was utilized to disclose whether melatonin may protect against ischemic neuronal death through microglia regulation. We found that melatonin promoted microglia/macrophage polarization toward anti‐inflammatory phenotype through the activation of STAT3 pathway and thereby inhibited the neurotoxic effect of pro‐inflammatory microglia to post‐OGD neurons.

## 2 MATERIALS AND METHODS

### 2.1 Animals

For the study, 8‐10 weeks‐old male C57BL/6J mice weighing 23‐25 g were purchased from the Vital River Laboratory Animal Technology Co., Ltd. All the animal experiments in this study were approved by the Institutional Animal Care and Use Committee of Capital Medical University and were performed in accordance with the National Institutes of Health Guidelines on the Care and Use of Laboratory Animals. A total of 36 mice were randomly assigned to three groups: (i) sham (6 mice); (ii) stroke with vehicle treatment (15 mice); and (iii) stroke with melatonin treatment groups (15 mice). All behavioral analysis was performed by independent investigators blinded to the treatment condition.

### 2.2 Distal middle cerebral artery occlusion model and melatonin treatment

Distal middle cerebral artery occlusion (dMCAO) model was induced according to our previous report.^15^ Briefly, anesthesia was induced with 2% isoflurane in N_2_/O_2_ (70%:30%) mixture. A ~ 2 cm skin incision was created between the right margin of the orbit and the tragus, and the temporalis muscle was dissected to expose right zygomatic arches and squamosal bone. A craniotomy was made, and the right middle cerebral artery (MCA) was exposed after the dura was opened. The right MCA distal to the lenticulostriate branches was occluded with bipolar electrocautery (Goldbov Photoelectronics CO. Ltd). Meanwhile, a 15 minutes bilateral occlusion of the common carotid artery (CCA) was performed. Rectal temperature was maintained at 37 ± 0.5°C using a heating pad (Harvard Apparatus). The sham mice were operated similarly, except for occlusion.

Melatonin (Sigma‐Aldrich) was dissolved in dimethylsulfoxide (DMSO) (Sigma‐Aldrich) and further diluted in saline. DMSO at the same concentration was used as vehicle control. Mice were intravenously injected with either melatonin (20 mg/kg) or the same volume of DMSO‐saline at 0 and 24 hours after reperfusion of the CCA.

### 2.3 Quantification of infarct volume

Infarct volume was measured at 3 days after ischemia on TTC‐stained sections. The mice were deeply anesthetized with chloral hydrate and decapitated. The brains were removed and coronally sliced to 1 mm sections in a brain mold. Then, the brain slices were incubated in 2% 2, 3, 5‐triphenyltetrazolium chloride (TTC; Sigma‐Aldrich) solutions at 37°C for 30 min. The infarct area was calculated as the area of the contralateral hemisphere minus the noninfarct area of the ipsilateral hemisphere using National institutes of Health ImageJ software. The infarct volume was obtained by the sum of the infarct areas in all sections multiplying by the slice thickness.

### 2.4 Adhesive removal test

An adhesive removal test was used to determine the sensorimotor deficits at 1 and 3 days after cerebral ischemia, as previously described.^15^ In brief, an adhesive tape (~ 50 mm^2^) was applied to the distal radial region of the right forelimb. The mice removed the tape using their left forelimbs in response to the tactile stimulations. The times to contact and to remove the tape were recorded. Each mouse was tested three times with a cutoff time of 120 seconds per trial. The data are presented as the mean times to contact or the mean times to remove the tape from three trials.

### 2.5 Modified garcia score test

The modified Garcia Score test was used to assess the behavioral function at 1 and 3 days after dMCAO. The modified Garcia Score system is composed of 5 tests:^15^ body proprioception, vibrissae touch, limb symmetry, lateral turning, and forelimb walking. The total score for the modified Garcia Score ranges from 0 (maximum deficits) to 15 (no deficits).

### 2.6 Pro‐inflammatory microglia cell model and melatonin treatment

BV2 (microglia cell line) and N2a (neuron cell line) were purchased from Cell Center, Institute of Basic Medical Sciences, Chinese Academy of Medical Sciences (CAMS), and Peking Union Medical College (PUMC). Both of them were cultured in the Dulbecco's Modified Eagle's Medium supplemented (Gibco) with 10% fetal bovine serum (Gibco) and 1% penicillin‐streptomycin (Gibco) at 37°C in CO_2_/air (5% /95%) mixture.^29^ We used OGD neuron‐conditioned medium (CM) to stimulate resting microglia. To induce ischemic injury, N2a cells were subjected to OGD for 3 hours and then returned to 95% air, 5% CO_2_, and normal glucose medium for 12 hours. To obtain pro‐inflammatory microglia, the OGD neuron CM (1:1 ratio to microglia media) was added to microglia cultures for 6, 12, or 24 hours. Control microglia were treated with medium collected from normal neuron without OGD. For dose‐response studies, IL‐1β mRNA level was measured using real‐time PCR after BV2 was stimulated by CM or CM plus melatonin (100, 200, or 400 mM) for 12 hours.

### 2.7 Neuron‐microglia co‐culture

BV2 microglia and N2a cells were cocultured using transwell cell culture inserts (Dow Corning, Corning). The cell density ratio of microglia to neurons is 1:10. Briefly, microglia growing on culture inserts were treated with CM followed by melatonin or vehicle for 12 hours. Medium was removed, and microglia washed with fresh medium 3 times. For N2a culture, neuronal cultures growing in 24‐well plate were subjected to OGD for 3 hours. Medium was removed, and neuron‐microglia co‐cultures were generated by adding the microglia inserts on top of post‐OGD neuronal cultures. The co‐culture was maintained for 12 hours before the microglia inserts were removed. Neuronal survival was measured by MTT kit (Roche) and cell death was quantified by a lactate dehydrogenase (LDH) release according to the manufacturer's protocol (Beyotime).

### 2.8 Real‐time PCR

Real‐time PCR was performed as described in our previous report.^15^ The total RNA from the ischemic brain or from the BV2 cells was isolated using TRIzol Reagent (Invitrogen) and was reverse‐transcribed into cDNA using by SuperScript II Reverse Transcriptase (Invitrogen) to detect gene expression according to manufacturer's instructions. Real‐time PCR was performed using quantitative PCR (ABI 7500, Thermo Fisher Scientific) with corresponding primers (Table 1, Invitrogen) in the presence of a fluorescent dye (RT2 SYBR® Green FAST Mastermixes, QIAGEN). Each experimental group was performed in triplicate to obtain the cycle time (CT) mean. GAPDH was used as a reference gene for quantification. The expression of mRNAs was presented as fold changes *vs*. sham control.

**Table 1:** Primers for real‐time PCR

Genes		Primers (5’‐3’)
GAPDH	Forward	CTCAAGATCATCAGCAATG
	Reverse	GTCATGAGTCCTTCCACG
IL‐1β	Forward	GTGGAACTTGAGGCCACATT
	Reverse	TGTGACAAAAATGCCTGGAA
TNF‐α	Forward	CAGGCGGTGCCTATGTCTC
	Reverse	CGATCACCCCGAAGTTCAGTAG
IL‐6	Forward	CCGGAGAGGAGACTTCACAG
	Reverse	TCCACGATTTCCCAGAGAAC
iNOS	Forward	TCCAGGATGAGGACATGAGCAC
	Reverse	GAACGTCACACACCAGCAGGTTA
CD11b	Forward	GACAGTGCTGGGAGACGTGAA
	Reverse	AGATCCTTACCCCCACTCAGAGA
CD86	Forward	TCTCCACGGAAACAGCATCT
	Reverse	CTTACGGAAGCACCCATGAT
CD206	Forward	CTCAACCCAAGGGCTCTTCTAA
	Reverse	AGGTGGCCTCTTGAGGTATGTG
TGF‐β	Forward	GCTGACAGAGGCACCACTG
	Reverse	CGCTGAATCGAAAGCCCTGTA
IL‐10	Forward	TGGCCCAGAAATCAAGGAGG
	Reverse	CAGCAGACTCAATACACACT
Arg1	Forward	CAGAAGAATGGAAGAGTCAG
	Reverse	CAGATATGCAGGGAGTCACC
Ym1/2	Forward	CAGGGTAATGAGTGGGTTGG
	Reverse	CACGGCACCTCCTAAATTGT

### 2.9 Western Blot

Cultured BV2 cells were used for Western blot analysis. Collected cells were lysed in RIPA buffer containing the protease and phosphatase inhibitors (Roche). The extracted proteins were quantified with bicinchoninic acid (BCA) kit (Thermo Fisher Scientific). Proteins were separated by 12% SDS‐PAGE gels and further transferred onto a PVDF membrane (Bio‐Rad). The membranes were blocked with 5% fat‐free milk in TBST for 1h at room temperature, and incubated with the primary antibodies, including signal transducer and activator of transcription 3 (STAT3, 1:5000, Abcam) and phospho‐STAT3 (p‐STAT3, 1:2000, Abcam) in TBST at 4°C overnight. Next, the membrane was incubated with the secondary antibody (1:5000, Abcam) for 1 hour at room temperature. All protein bands were visualized by ECL detection systems (Bio‐Rad). GAPDH (Abcam) was used as an internal control. The intensities of protein bands were analyzed densitometrically using Image J.

### 2.10 Statistical analysis

The number of mice in each group was determined using power analyses, according to our past experience with similar measurements (α = 0.05 and β = 0.20). All data were reported as mean ± SEM. Comparison of means between two experimental groups was evaluated by the Student's two‐tailed t‐test. Differences in means among multiple groups were analyzed using one‐ or two‐way analysis of variance followed by the Bonferroni test. Statistical significance was set at *P* < .05.

## 3 RESULTS

### 3.1 Poststroke administration of melatonin reduces infarct volume and improves sensorimotor functions after dMCAO

**Figure 1:**
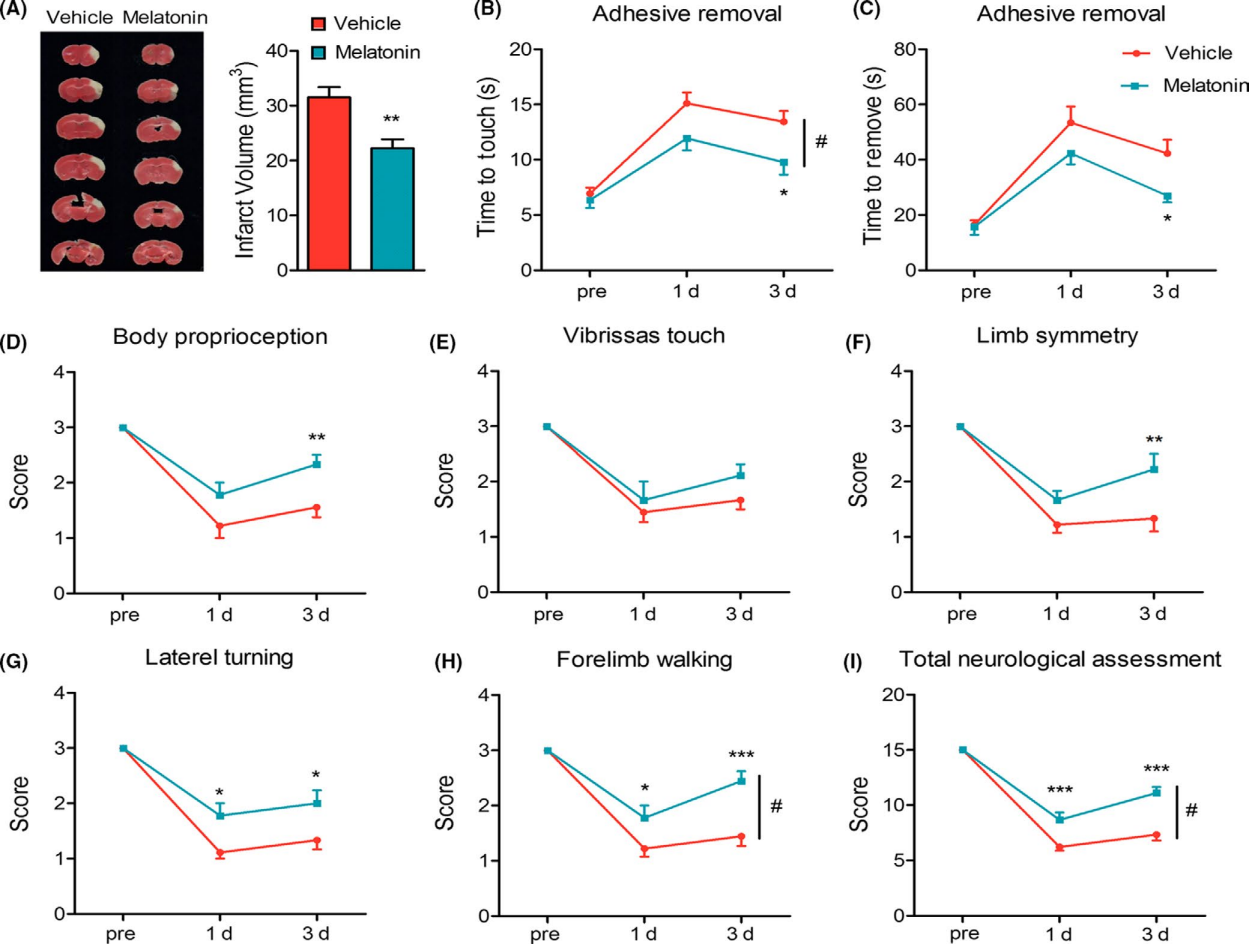
Melatonin reduces infarct volume and improves sensorimotor functions early after dMCAO. Mice were subject to dMCAO and received melatonin (20 mg/kg) injections at 0 and 24 hours after dMCAO. A, Representative TTC staining and quantification of infract volume 3 days after dMCAO. B,C, Adhesive removal test. Both time to touch (B) and time to remove (C) were recorded. D,I, Garcia scores were used to evaluate sensorimotor deficits at pre‐surgery, 24, 72 hours after operation, including (D) body proprioception, (E) vibrissae touch, (F) limb symmetry, (G) lateral turning, (H) forelimb walking, and (I) total neurologic assessment score. Data are mean ± SEM. N = 9/group. *P < .05, **P < .01, ***P < .001 between vehicle and melatonin group. Two‐way repeated measures ANOVA followed by Bonferroni post hoc test. #*P* < .05 *vs* vehicle group

Mice were subjected to dMCAO and then to receive injections of either melatonin (*i.p.*, 20 mg/kg) or vehicle at the onset of cerebral ischemia and 24 hours after dMCAO. As shown (Figure 1A), melatonin significantly reduced infarct volume 3 days after dMCAO. To determine the effect of melatonin treatment on functional outcomes after dMCAO, sensorimotor deficits were evaluated by the adhesive removal and modified Garcia score system 1 and 3 days after ischemia. Melatonin treatment markedly reduced the time to touch the tape (Figure 1B) and the time to remove the tape (Figure 1C) 3 days after stroke as compared to vehicle‐treated mice. Moreover, administration of melatonin reversed the stroke‐induced sensorimotor deficits in body proprioception (Figure 1D), limb symmetry (Figure 1F), lateral turning (Figure 1G), and forelimb walking (Figure 1H), resulting in improved total neurological scores on days 1 and/or 3 after stroke (Figure 1I). There was no significant difference in vibrissae touch (Figure 1E) between vehicle and melatonin‐treated groups. These results indicate that melatonin treatment improved the short‐term behavioral functions after dMCAO.

### 3.2 Melatonin shifts microglia/macrophage polarization toward anti‐inflammatory phenotype 3 days after dMCAO

**Figure 2:**
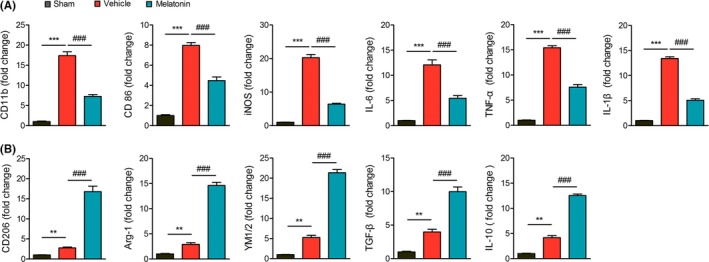
Melatonin shifts microglia/macrophage polarization toward anti‐inflammatory phenotype 3 days after dMCAO. Mice were subject to dMCAO and received melatonin (20 mg/kg) injections at 0 and 24 hours after dMCAO. Peri‐infarct areas of brains were collected at 3 days after dMCAO for RNA preparation. A, mRNA expression of pro‐inflammatory genes (CD11b, CD86, iNOS, TNF‐α, IL‐6, and IL‐1β) was measured by real‐time PCR. B, mRNA expression of anti‐inflammatory genes (CD206, Arg‐1, YM1/2, TGF‐β, and IL‐10) was measured by real‐time PCR. Data are mean± SEM. n = 6/group, **P < .01, ***P < .001 *vs* sham; ###*P* < .001 *vs* vehicle group, one‐way ANOVA followed by Bonferroni post hoc test

The polarized microglia/macrophages are commonly distinguished by their expression of feature genes. We measured the mRNA expression of microglia/macrophage phenotype markers in the ischemic brain using real‐time PCR at 3 days after dMCAO (Figure 2). Our data show that the expression of pro‐inflammatory phenotype markers (CD11b, CD86, iNOS, IL‐6, and TNF‐α) and anti‐inflammatory phenotype markers (CD206, Arg‐1, YM1/2, TGF‐β, and IL‐10) was all increased greatly after dMCAO. However, the pro‐inflammatory markers increased much more than anti‐inflammatory markers. Melatonin treatment enhanced the expression of above‐mentioned anti‐inflammatory markers while inhibited the expression of pro‐inflammatory markers in the ischemic brain at 3 days after dMCAO.

### 3.3 Melatonin promotes microglia polarization toward anti‐inflammatory state and alleviates the pro‐inflammatory responses in vitro

**Figure 3:**
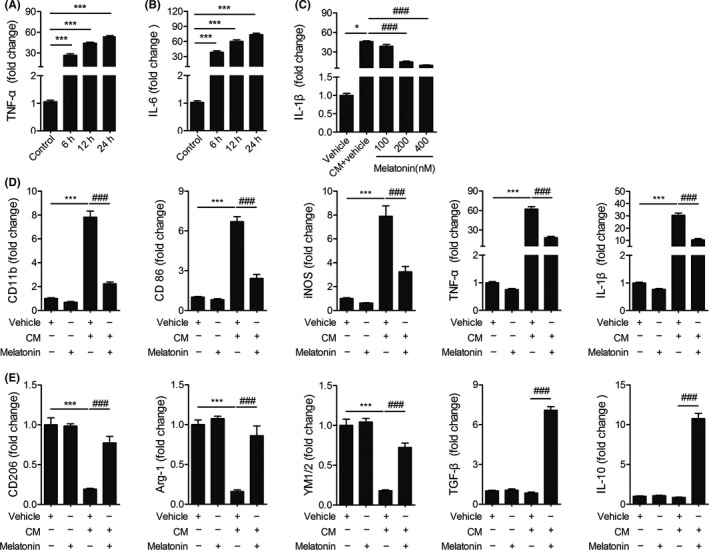
Melatonin inhibits inflammation responses in microglia. Microglia BV2 cells were treated with conditioned medium (CM) collected from OGD‐challenged neuronal cell line N2a, together with melatonin or vehicle (ethanol). A,B, mRNA expression of pro‐inflammatory genes (TNF‐α (A), IL‐6 (B)) was measured at 6, 12, 24 hours after stimulation with CM. *P < .05, ***P < .001 vs control group, one‐way ANOVA followed by Bonferroni post hoc test. C, mRNA expression of IL‐1β was measured at 12 hours after stimulation with CM together with melatonin (100, 200, 400 nM). D, mRNA expression of pro‐inflammatory microglia/macrophage signature genes (CD11b, CD86, iNOS, TNF‐α, and IL‐1β) was measured by real‐time PCR at 12 hours after exposure to OGD neuron CM, together with melatonin (200 nM) or vehicle. E, mRNA expression of anti‐inflammatory microglia/macrophage signature genes (CD206, Arg‐1, YM1/2, TGF‐β, and IL‐10) was measured by real‐time PCR. Data are mean ± SEM. n = 3/group, *P < .05, ***P < .001 *vs* vehicle group; ###*P* < .001 *vs* CM + vehicle group, one‐way ANOVA followed by Bonferroni post hoc test

To further determine the effect of melatonin on microglia polarization and inflammatory responses, we tested the changes in a battery of phenotype markers in BV2 cells in response to exposure to CM collected from post‐OGD neuron. The gene expressions of the pro‐inflammatory cytokines (TNF‐α and IL‐6) were remarkably increased at various time points (6, 12, or 24 hours) after the CM stimulation (Figure 3A‐3B). We chose 12 hours as the time point for later experiments.

We observed that the mRNA expression of IL‐1β in BV2 cells was also significantly increased 12 hours after OGD neuron CM treatment (Figure 3C). Melatonin at 200 or 400 nM inhibited CM‐induced IL‐1β production in BV2 cells. There was no significant difference in IL‐1β level between 200 and 400 nM treated groups. Therefore, 200 nM was used as the optimal dose for later experiments.

We further found that the mRNA expression of pro‐inflammatory microglia marker (CD11b, CD86, iNOS, TNF‐α, and IL‐1β) was increased significantly in BV2 cells 12 hours after stimulated with CM from post‐OGD neurons, and melatonin (200 nM) markedly ameliorated such inflammation induction. Moreover, melatonin treatment revered the reduction of mRNA expression of anti‐inflammatory microglia marker (CD206, Arg‐1, YM1/2) in CM‐stimulated BV2 cells. These in vitro results are in accordance with the in vivo studies. No dramatic alterations in the mRNA expression of anti‐inflammatory cytokines (IL‐10 and TGF‐β) were observed in BV2 after stimulation with CM compared with nonstimulated group (Figure 3E). It should be noted that melatonin treatment enhanced the mRNA expressions of these two mediators in CM‐stimulated BV2. Taken together, our in vivo and in vitro data show that melatonin exerted a potent regulatory effect on microglia polarization, promoting anti‐inflammatory polarization and inhibiting pro‐inflammatory responses after stimulation.

### 3.4 Melatonin regulates microglia polarization toward pro‐inflammatory phenotype through STAT3 pathway

**Figure 4:**
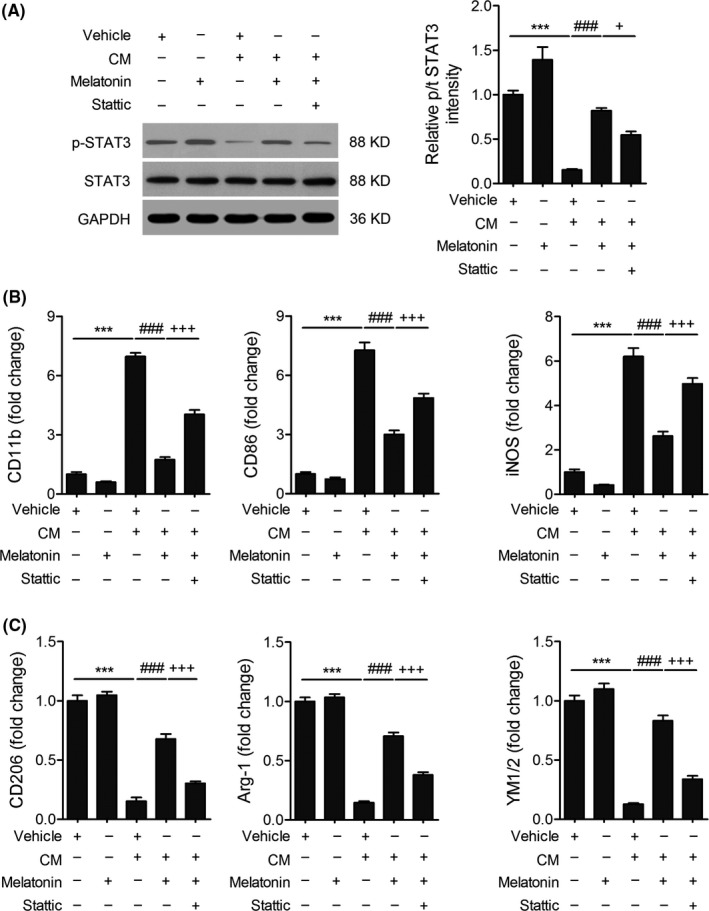
Melatonin shifts microglia polarization *via* the activation of STAT3 signaling pathway. A, Representative Western blots and quantification of p‐STAT3 and STAT3 in the BV2 microglia treated OGD neuron CM with/without 200 nM melatonin. STAT3 inhibitor stattic was used as a negative control. B,C, The importance of STAT3 in the effect of melatonin on microglia polarization. Stattic (5 μM) was added to OGD‐treated microglia for 12 hours. B, mRNA expression of anti‐inflammatory microglia/macrophage signature genes (CD11b, CD86, and iNOS) was measured by real‐time PCR. C, mRNA expression of pro‐inflammatory microglia/macrophage signature genes (CD206, Arg‐1, YM1/2) was measured by real‐time PCR. Data are mean ± SEM. n = 3/group, ***P < .001 *vs* vehicle group; ###*P* < .001 *vs* CM + vehicle; +*P* < .05, +++*P* < .001 *vs* CM + melatonin group, one‐way ANOVA followed by Bonferroni post hoc test

STAT3 plays a critical role in macrophage polarization.^30^, ^31^ In order to elucidate the mechanism underlying melatonin's regulation on microglia polarization, we examined the protein levels of STAT3 and p‐STAT3 in BV2 cells. The ratio of p‐STAT3/STAT3 expression was decreased in pro‐inflammatory microglia stimulated by CM compared with resting microglia, whereas melatonin treatment reversed this alteration. Administration of melatonin dramatically upregulated the relative expression of *p*‐STAT3/STAT3 (Figure 4A). To further clarify the underlying role of STAT3 pathway, stattic was used to inhibit activation of STAT3. We found that the mRNA expressions of pro‐inflammatory phenotype markers were increased, whereas the mRNA expressions of anti‐inflammatory phenotype markers were decreased when stattic was added to CM‐stimulated BV2 cells in the presence of melatonin (Figure 4B,C). These results indicate that melatonin shifts microglia from pro‐inflammatory phenotype to anti‐inflammatory phenotype polarization state, at least in part, through STAT3 activation.

### 3.5 Melatonin reduces the neurotoxic effect of pro‐inflammatory microglia on post‐OGD neurons

**Figure 5:**
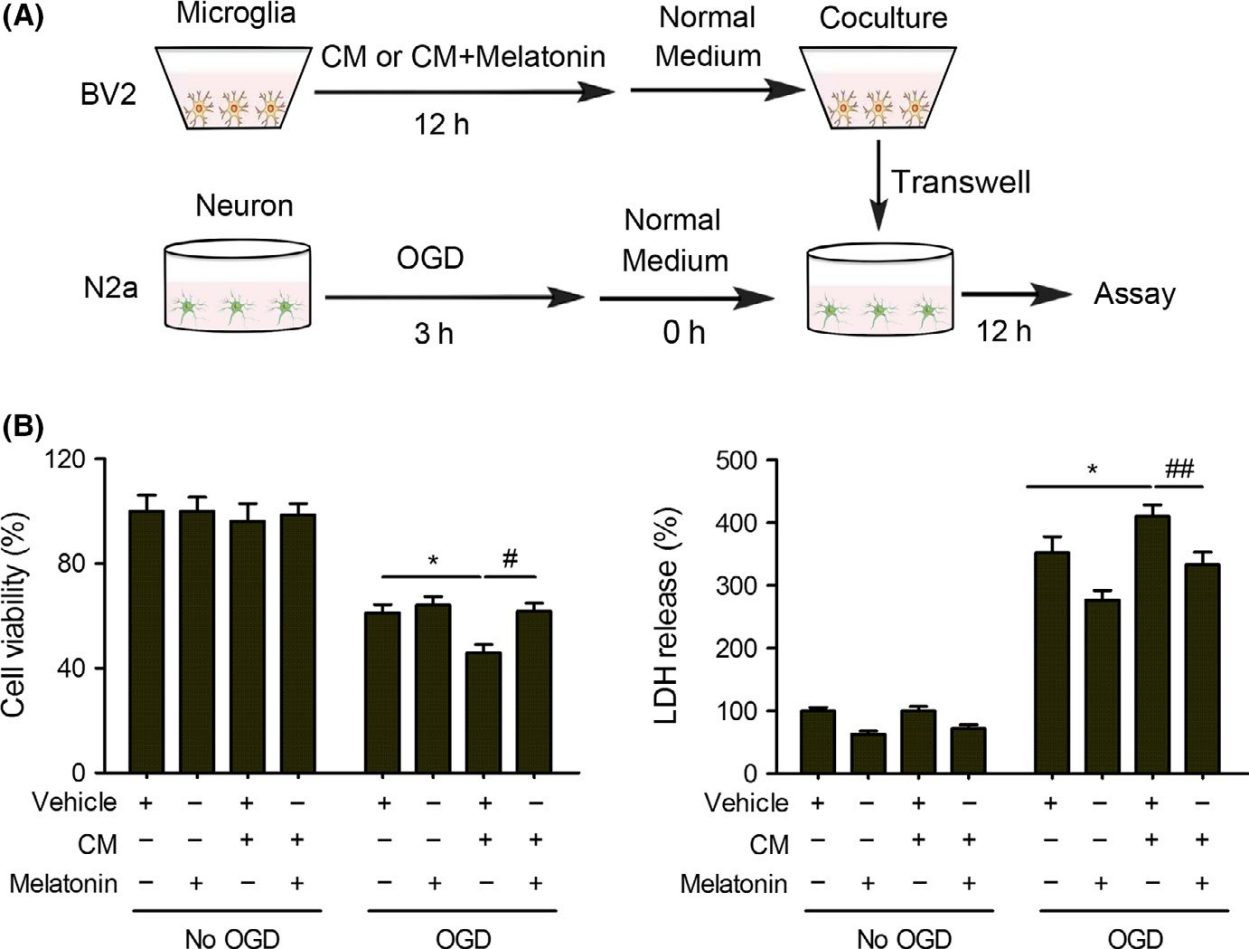
Melatonin abolishes the neurotoxic effect of pro‐inflammatory microglia in cocultured neurons. A, Microglia BV2 culture in transwell was exposed to regular microglia media (M0), or conditioned medium collected from OGD N2a (CM) (M1) for 12 hours in the absence or presence of melatonin (200 nM). N2a culture was subjected to OGD for 3h. Microglia in transwell was applied over the non‐OGD or post‐OGD neuronal N2a cultures for 12 hours. B, N2a survival was quantified by MTT assay. C, Cell death was quantified by LDH release. Data are mean ± SEM. n = 3/group, *P < .05 *vs* vehicle group; #*P* < .05, ##*P* < .01 *vs* CM + vehicle, one‐way ANOVA followed by Bonferroni post hoc test

We demonstrate that poststroke treatment of melatonin increased the expression of anti‐inflammatory phenotype markers and reduced the expression of pro‐inflammatory phenotype markers in the brain at 3 days after dMCAO, which is accompanied by reduced cerebral inflammation and infarct volume (Figure 1 and Figure 2). To further confirm that melatonin could indirectly protect against ischemic neuronal death through microglia regulation, a microglia‐neuron co‐culture system was used (Figure 5A). Pro‐inflammatory phenotype microglia enhanced post‐OGD neuronal death, as indicated by increased LDH release and decreased cell viability results, which was significantly reversed in melatonin‐treated CM (Figure 5B,C). These findings suggest that melatonin converted microglia polarization toward anti‐inflammatory phenotype and attenuated pro‐inflammatory phenotype–potentiated neurotoxic effect on ischemic neuronal death.

## 4 DISCUSSION

Melatonin is a natural neurohormone produced mainly in pineal gland and is a powerful antioxidant, free radical scavenger, and a regulator of circadian rhythm.^32^ The decreased melatonin levels were observed in acute ischemic stroke patients or in the experimental stroke models.^33^ Accumulating evidence has endorsed that exogenous melatonin treatment plays a neuroprotective role in several CNS disorders, including ischemic stroke, through the mechanism of elimination free radical, regulation of circadian rhythm, inhibition of inflammatory responses.^24^ In this study, a mouse distal middle cerebral artery occlusion model is used to demonstrate the protective effect of melatonin. We found that melatonin significantly reduced infarct volume and remarkably improved several aspects of sensorimotor function during the acute phase (3 days) of ischemic stroke. Melatonin treatment was reported to improve long‐term functional outcome after transient focal cerebral ischemia.^34^ The cellular mechanism of melatonin‐afforded protection can be multifaceted, including maintaining Ca^2+^ homeostasis,^21^ suppressing inflammatory response,^28^ decreasing oxidative stress,^35^ modulating stem cell survival,^35^, ^36^ and attenuating endoplasmic reticulum stress.^37^ In the present study, we specifically evaluated the effect of melatonin in the modulation of microglia phenotypic responses.

Microglia/macrophages are plastic cells with different phenotypes that can be involved in distinct functions. Consistent with previous study, stroke enhanced both pro‐inflammatory and anti‐inflammatory markers at 3 days post‐dMCAO. Administration of melatonin significantly reduced pro‐inflammatory markers but greatly increased anti‐inflammatory markers, accompanied by reduced brain infarct. For example, we observed elevated expression of IL‐10, a beneficial cytokine with inhibition on inflammation and immune reaction,^38^ at 3 days after dMCAO. Interestingly, melatonin treatment further increased the expression of IL‐10 compared with vehicle‐treated group. Similarly, higher level of anti‐inflammatory cytokine TGF‐β was observed in melatonin‐treated dMCAO mice. These results can be replicated in OGD neuron CM–treated microglia, suggesting that melatonin treatment can help to reduce the pro‐inflammatory response and increase anti‐inflammatory response in microglia upon ischemic neuronal injury. Furthermore, our in vitro studies confirmed that melatonin reduced the neurotoxic effect of pro‐inflammation microglia on post‐OGD neurons. It is known that microglia/macrophage polarization could be regulated by cytokine‐dependent microenvironments. For instance, interferon γ (IFN‐γ) and TNF‐α can induce and maintain pro‐inflammation microglia polarization, whereas IL‐4 or IL‐10 can initiate anti‐inflammatory polarization.^39^, ^40^, ^41^ Therefore, the reduced inflammatory mediator release after melatonin treatment could improve microenvironment and further ameliorate inflammation and/or promote brain recovery.

Microglia/macrophage polarization is modulated by many transcription factors, such as STATs and PPAR‐γ^6^, ^42^, ^43^ STAT3 was demonstrated to play a dominant role in IL‐10‐mediated anti‐inflammatory effects in human macrophages.^8^ In addition, STAT3 appeared to be critical in regulating macrophage/microglia polarization to anti‐inflammatory phenotype in the TBI brain.^30^ Besides, resveratrol was reported to suppress the LPS‐induced pro‐inflammatory response in microglia cells through the activation of STAT3 pathway.^31^ In this study, melatonin significantly increased the expression of phosphorylated STAT3 in microglia. Melatonin‐induced anti‐inflammatory polarization was abolished by the STAT3 inhibitor, stattic. These results further illustrated that melatonin regulates microglia polarization, at least in part, through STAT3 pathways. Here, we reveal the role of STAT3 in melatonin‐induced anti‐inflammatory polarization in cultured microglia. Nevertheless, in vivo study is still required to perform to confirm the underlying mechanism.

## 5 CONCLUSION

We demonstrate that melatonin has a profound regulatory effect on microglia/macrophage polarization toward anti‐inflammatory phenotype through STAT3 pathway. Our findings suggest that melatonin post‐treatment reduces ischemic stroke–induced brain damage and improves functional outcomes, providing new evidence that melatonin might be a promising therapeutic strategy for stroke.

## CONFLICT OF INTEREST

The authors declare no conflict of interest.
